# Novel leaderless bacteriocin geobacillin 6 from thermophilic bacterium *Parageobacillus thermoglucosidasius*

**DOI:** 10.3389/fmicb.2023.1207367

**Published:** 2023-06-15

**Authors:** Ana Koniuchovaitė, Akvilė Petkevičiūtė, Emilija Bernotaitė, Alisa Gricajeva, Audrius Gegeckas, Lilija Kalėdienė, Arnoldas Kaunietis

**Affiliations:** Department of Microbiology and Biotechnology, Institute of Biosciences, Life Sciences Center, Vilnius University, Vilnius, Lithuania

**Keywords:** leaderless bacteriocin, thermophilic bacteria, geobacillin, *Parageobacillus*, heterologous expression

## Abstract

Bacterial resistance to conventional antibiotics has urged us to develop alternative strategies against bacterial pathogens. Moreover, a demand for food products containing no chemical preservatives has led us to search for new alternative technologies for food preservation. Bacteriocins – ribosomally synthesized antimicrobial peptides – have been proposed as a new alternative to conventional antibiotics or chemicals for food preservation. This study describes biosynthesis and characterization of a novel leaderless bacteriocin, geobacillin 6, which was identified in the thermophilic bacterium *Parageobacillus thermoglucosidasius.* Its amino acid sequence shows low similarity to other bacteriocins and it is the first leaderless-type bacteriocin identified in thermophilic bacteria. Based on structure assessment, the bacteriocin forms a multi-helix bundle. Geobacillin 6 exhibits a relatively narrow antimicrobial spectrum, it is active in the μM range and against Gram-positive bacteria, mostly thermophilic species closely related to the producer strain. Bacteriocin demonstrates stability over pH 3–11 and is highly thermostable, retaining 100% of its activity after incubation at 95°C for 6 h. Geobacillin 6 has potential in the food industry and biotechnological processes where contamination with thermophilic bacteria is undesirable.

## 1. Introduction

Bacterial resistance to conventional antibiotics has dramatically increased. Multidrug-resistant opportunistic microorganisms are spreading and today it is a major threat to public health. New antibacterial drugs against global priority pathogens have been developed, but they are derived from already established antibiotic classes. Unfortunately, only a few antibacterial agents of novel classes have been discovered to date. For this reason, scientists are urged to develop and propose new alternative strategies against bacterial pathogens. Moreover, the increasing demand for chemical-free food products and the potential health hazards of artificial additives used in food preservation have led to a search for alternative technologies for the preservation of food products (Cotter et al., 2005, 2013; Global Action Plan on Antimicrobial Resistance, 2015).

Bacteriocins are ribosomally synthesized antimicrobial peptides or small proteins produced by bacteria. These antimicrobials have an antagonistic activity against other bacteria meanwhile the producer has immunity to the produced bacteriocin. Bacteriocins are very diverse in their size, structure, mode of action, or immunity mechanisms. These peptides can target different receptors and have broad or narrow antimicrobial spectrum (Soltani et al., 2021).

Bacteriocins can have antimicrobial activity against multi-drug resistant pathogens and biopreservation using these antimicrobial peptides as an alternative to conventional antibiotics is a promising area. Because they are thermostable, retains stability in a wide pH range, have no color and odor, and can be inactivated by proteolytic enzymes, bacteriocins can be used as a biotechnological tool in the food and pharmaceutical industries (Bhattacharya et al., 2022). To date, only one of bacteriocin, nisin synthesized by *Lactococcus lactis*, is licensed as a biopreservative and is exploited in the food industry (Subramanian and Smith, 2015). Recently, in the United States through the GRAS regulatory procedure, some recombinant bacteriocins produced in plants have received promising regulatory reviews for application as food antibacterials (Hahn-Löbmann et al., 2019). In addition to their potential use in the medicine and food industries, these antimicrobials have perspectives for use in food animals and aquaculture, as therapeutics against bacterial infections. Thus, bacteriocins represent feasible alternatives to antibiotics (Pereira et al., 2022).

Studies on bacteriocins produced by extremophiles, like thermophilic bacteria, are very limited. Most of these antimicrobials are described very obscurely and most importantly, the amino acid sequences have not been revealed and provided. Usually, they are described as bacteriocin-like substances. To date, only four bacteriocins from thermophilic bacteria have been identified and well-characterized (Garg et al., 2012; Egan et al., 2018; Kaunietis et al., 2019; Vaičikauskaitė et al., 2019). Thus, the group of bacteria is an unexplored and rich source of novel bacteriocins. Thermophiles can reveal new and promising antibacterial agents with useful properties such as high thermostability.

In canned food and dairy industries contamination with thermophilic spore-forming microorganisms is responsible for a ‘flat’ sour spoilage of canned foods and products like evaporated milk. If the temperature is suitable, thermophilic bacteria can grow and form biofilms in diary manufacturing plants, potentially causing contamination. This can lead to product spoilage due to the production of acids and enzymes (Kalogridou-Vassiliadou, 1992; Watterson et al., 2014; Burgess et al., 2015; Seale et al., 2015; Berendsen et al., 2016). Thermophilic microorganisms are utilized in industrial-level bioethanol or lactate production. However, contamination with other thermophiles during these processes is also possible (Cripps et al., 2009; Bacon et al., 2017). Bacteriocins active against thermophiles could be used to avoid contamination of thermophilic spore-forming bacteria in these industry processes.

This study reveals novel antimicrobial peptide geobacillin 6 encoded in thermophilic bacteria *Parageobacillus thermoglucosidasius* DSM 2542. This peptide represents a small subclass of leaderless bacteriocins, which compared to other bacteriocins do not have leader sequences and require no post-translational modifications to obtain antimicrobial activity (Masuda et al., 2012).

## 2. Methods

### 2.1. Bioinformatics analysis

The genome sequences of *P. thermoglucosidasius* DSM 2542 (GenBank: LAKX00000000) were derived from the NCBI database at.^1^ Bacteriocin gene mining in the genome of *P. thermoglucosidasius* DSM 2542 was performed by BAGEL4 web-server at.^2^ Prediction of signal sequence in bacteriocin was performed by SignalP 5.0 Server at.^3^ BLAST protein sequence analysis was performed using the NCBI database at^4^ and the UniProt database at.^5^ Protein sequence alignment was performed using the UniProt database at.^6^ Protein structure prediction was performed by the I-TASSER web server at.^7^ To evaluate the secondary structure of the peptide a DichroWeb online analysis was performed on a web-server at.^8^

### 2.2. Growth media and cultivation conditions

For routine cultivation of thermophilic bacteria CASO broth (Roth) or NB medium was used and supplemented with 15 g/L agar (Oxoid) when needed. For NB medium preparation 10 g/L of tryptone from casein (Roth), 5 g/L of meat extract, and 5 g/L of NaCl (Roth) were used. For mesophilic bacteria cultivation LB or LB-agar (Biolab) media were used. Yeasts were grown in YPD medium, which contained 2% peptone from casein (Roth), 1% yeast extract (Roth), and 2% glucose (Roth) and supplemented with 15 g/L agar (Fisher Scientific) when needed. Medium selection for each strain in experiments is listed in Supplementary File 1.

Growth media supplemented with glycerol [20% (w/w)] were used as stock culture stored at −75°C temperature. Mesophilic bacteria strains were cultivated in a thermostat or thermoshaker with aeration at 37°C, thermophilic bacteria at – 55°C, and yeasts at – 30°C.

### 2.3. Antimicrobial activity assays

For the spot on a lawn assay using mesophiles, an overnight culture of the indicator strain was prepared by cultivating it at 30/37°C. Then, 1% of the culture was inoculated into melted agar medium and poured into Petri plates. Once the medium solidified, 10 μl of the bacteriocin sample was spotted on the plate. The plate was then incubated overnight in a thermostat at 30/37°C. The next day, the growth inhibition zone of the indicator strain was evaluated on the plate.

For the antimicrobial activity assay using thermophiles, a fresh colony of the indicator strain was inoculated into a liquid medium and cultivated overnight at 55°C in a thermoshaker. The following morning, the culture was inoculated (2–3%) into fresh medium and cultivated until the optical density (OD) reached 1–1.5. Then, the culture was mixed with melted agar medium at a ratio of 1:10 and poured into Petri plates.

For the spot on a lawn assay, 10 μl of the sample was spotted on the plate. To compare the activity between different samples, serial two-fold dilutions of the samples were made and dispersed on the plate. The arbitrary unit of antibacterial activity per milliliter (AU/ml) was defined using a formula 2^n^ × 1 ml/V, where V represents the sample volume (ml) and n represents the titer of the reciprocal highest dilution that results in growth inhibition of the indicator strain (Daba et al., 1991). Next, the plate was incubated overnight at 55° in a thermostat. The following day, the growth inhibition zone of the indicator strain was evaluated on the plate. A list of bacteria and yeast strains with their respective cultivation conditions can be found in Supplementary File 1.

### 2.4. Reagents for DNA extraction and cloning

DNA amplification was conducted using Phusion HF DNA polymerase, while genomic DNA extraction was performed using the GeneJET Genomic DNA Purification Kit. For plasmidic DNA extraction, the GeneJET Plasmid Miniprep Kit was used. Amplified or digested DNA were cleaned with the GeneJET PCR Purification Kit. For DNA cloning T4 DNA Ligase enzyme was used. For DNA digestion restriction endonucleases FastDigest NcoI and FastDigest BamHI were used. DNA extraction or amplification was confirmed by 1% agarose gel electrophoretic analyses. All reagents and kits mentioned in this section were purchased from Thermo Fisher Scientific and used according to manufacturers’ recommendations.

### 2.5. Cloning of DNA coding *His-TEV-geo6A* gene into a vector

A synthetic DNA coding Geo6A precursor peptide with optimized codon sequences for *E. coli* was ordered using GeneArt Strings DNA Fragments (Thermo Fisher Scientific) biosynthesis service. The DNA sequence was designed (Supplementary File 2) to code His6-tag and TEV peptidase recognition sites at the N-terminus of the peptide. DNA fragment *His-TEV-geo6A* was ligated into vector pET15b between NcoI and BamHI restriction sites using T4 DNA ligase and restriction endonucleases. The ligated DNA was transformed into *E. coli* DH5α by electroporation. Positive transformants were selected on LB-agar plates containing 50 μg/mL ampicillin and by colony PCR using Duet-up1 (5′-GGATCTCGACGCTCTCCCT-3′) and T7 Terminator (5′-GCTAGTTATTGCTCAGCGG-3′) primers. The constructed vector containing cloned gene (pET-His-TEV-geo6A) was extracted from bacteria culture and the DNA sequence of the insert was confirmed by Sanger sequencing.

### 2.6. Heterologous expression of *His-TEV-geo6A* gene

*E. coli* BL21 (DE3) cells were transformed with pET-His-TEV-geo6A vector and inoculated to liquid LB medium containing 50 μg/ml ampicillin and cultivated in a thermoshaker. The overnight culture was inoculated (1%) to fresh medium with antibiotics and grown until OD reached 0.9–1.2. Then gene expression was induced using IPTG 1 mM end concentration. After 4 h of cultivation in thermoshaker bacterial culture was collected by centrifugation at 5,000 ×*g* for 20 min at 4°C. Harvested cells were resuspended in buffer A (25 mM sodium phosphate, 500 mM NaCl, pH 7.4) and kept at −75°C temperature until further use.

### 2.7. Purification of bacteriocin

After induction harvested cells were sonicated on ice for 20 min using ultrasound sonicator VCX 130 (Sonics and Materials) with cycle 5 s ON and 10 s OFF and using 35% amplitude. Cell debris was separated by centrifugation at 15,000 ×*g* for 20 min at 4°C. The supernatant containing soluble fraction was diluted with binding buffer A up to 100 ml and filtered using a 0.45 μm filter. Further protein purification was performed using the BioLogic DuoFlow chromatography system (Bio-Rad) and the sample was loaded on Ni^2+^ affinity chromatography column HisTrap HP 1 ml (GE) previously equilibrated in the buffer A (section 2.6). After sample application, the column was washed with buffer B (25 mM sodium phosphate, 500 mM NaCl, 20 mM imidazole, pH 7.4). Protein was eluted using a step gradient with buffer C (25 mM sodium phosphate, 500 mM NaCl, 500 mM imidazole, pH 7.4). Elution fractions containing peptides were collected and further loaded on HiTrap HP 5 ml (GE) desalting column using buffer D (50 mM sodium phosphate, 100 mM NaCl, pH 7.4). Next, elution fractions of desalted protein were mixed with TEV protease, which was made in our laboratory according to Kapust et al. (2001). The reaction was performed in buffer D containing 5 mM DTT and incubated in a water bath for 4 h at 37°C temperature. After treatment with protease sample was diluted in buffer E (20 mM sodium phosphate, pH 7.4) and loaded on cation exchange column UNO S-1 (Bio-Rad) previously equilibrated in the same buffer D. Further, the column was washed with buffer D and protein was eluted using linear grading with buffer E (20 mM sodium phosphate, 1 M NaCl, pH 7.4). Elution fractions were collected and analyzed using tricine-SDS-PAGE (section 2.8).

### 2.8. Tricine-SDS-PAGE analysis

Cell samples or elution samples were analyzed by tricine-SDS-PAGE (Schägger, 2006). The electrophoresis was carried out using 4% stacking and 16% separating polyacrylamide gels. Following protein electrophoresis, the gel was stained using PageBlue Protein Staining Solution (Thermo Fisher Scientific) as per the manufacturer’s instructions.

### 2.9. Bacteriocin thermostability and stability in different pH ranges

For thermostability assay purified recombinant Geo6 was incubated at different temperatures: 60°C, 80°C and 100°C for 2 h and at 95°C up to 6 h. After incubation, serial two-fold dilutions of samples were made using medium, and further antibacterial activity of bacteriocin was assessed by spot on a lawn assay.

For evaluation of stability at different pH ranges, purified recombinant Geo6 was mixed with 25 mM Britton-Robinson buffer solutions (Ebihara et al., 2016) with different pH values in a ratio of 1:20. After incubation at room temperature for 2 h samples were neutralized with NaOH or HCl solutions to pH 7. Further, samples were diluted two-fold using a medium and assessed for antibacterial activity using spot on a lawn assay.

### 2.10. Minimum inhibitory concentration assay

MICs of Geo6 were determined according to the method of Wiegand et al. (2008) with some modifications. An indicator strain sensitive to bacteriocin was grown overnight at 55°C in a thermoshaker. The next day it was inoculated (2–3%) into fresh medium and cultivated until OD reached 1–1.5. The culture was further diluted and adjusted to 1 × 10^6^ CFU/ml. A 95 μl of the medium was mixed with 5 μl of Geo6 and serial two-fold dilutions of suspension were made with the medium. Next, diluted mixtures were dispersed (100 μl) in a 96-well plate. A volume of 100 μl of previously prepared cell suspension was dispersed and mixed with the diluted bacteriocin in every well on the plate. As a result, the final volume of suspension in the well containing diluted bacteriocin and bacteria cells was 200 μl and the final concentration of the bacteria culture in the well was 5 × 10^5^ CFU/ml. For each experiment, three replicates were made in the plate. Wells with positive controls contained a mixture of medium (95 μl) and Geo6 (5 μl), and negative controls contained bacteria suspension (5 × 10^5^ CFU/ml) in the medium (100 μl). The plate was placed into a bag to prevent medium evaporation and put into a box, which was incubated overnight at 55°C in a thermoshaker. The next day, the growth inhibition was evaluated in the wells of the plate.

### 2.11. Live/dead assay

An indicator strain *G. kaustophilus* HTA 426^T^ sensitive to bacteriocin was grown in BHI medium at 55°C till OD 0.5 was reached. Next, the bacteria culture was treated with 600 nM of Geo6 and further cultivated for 30 min. Then, the cell culture was centrifuged at 3000 ×*g* for 5 min and further washed two times with 0.9% NaCl solution (centrifuged at 3000 ×*g* for 5 min). After the last wash, cells were suspended in 0.9% NaCl solution and stained using LIVE/DEAD BacLight Bacterial Viability Kit (Invitrogen) containing SYTO9 and fluorescent dyes according to manufacturers’ recommendations. Cells were incubated in the dark at room temperature for 15 min. Next, cells were centrifuged at 3000 ×*g* for 5 min and washed two times with 0.9% NaCl solution (centrifuged at 3000 ×*g* for 5 min). Further, 10 μl of Geo6 treated cells and nontreated control cells suspensions were spotted onto objective slides and examined using a fluorescence microscope using filters FITC (480/500 nm) and Texas Red (490/635 nm).

### 2.12. Circular dichroism spectroscopy

Peptide solution was prepared in 10 mM sodium phosphate buffer (pH 7.4) and at a final concentration of 10 μM. The circular dichroism (CD) data was collected using a J-810 CD Spectrophotometer (JASCO) in a Cuvette of 0.125 cm path length and in a range of 190–280 nm at 0.5 nm intervals. The spectrum of the scan was recorded with a 2 nm optical bandwidth and presented as the average of 4 scans. Baseline measurements were recorded using a buffer solution and then subtracted from the sample scan.

### 2.13. Geo6 thermal shift assay

As an indicator of thermal denaturation in varying pH or other conditions a fluorescent SYPRO Orange dye (Invitrogen) that binds to hydrophobic parts of protein during its unfolding was used in this work. SYPRO Orange 50× stock solution was prepared by diluting 2.5 μl of 5,000× concentrate into 250 μl of deionized (DI) water. A volume of 2.5 μl of 50× SYPRO Orange was aliquoted into a white HardShell 96-well PCR plate (Bio-Rad). Different pH buffer solutions (25 mM Briton-Robinson, pH 3–11; Bendikienė et al., 2004) were added to aliquoted SYPRO Orange followed by adding an appropriately diluted solution of purified Geo6 into the wells at a final concentration of 0.25 nmol/ml (1.3 μg/ml). The final volume of the mixture was 25 μl. The negative control was 25 mM of Briton-Robinson buffer solutions in a range of pH 3 to 11, with 2.5 μl of SYPRO Orange. Lysozyme (final concentration of 100 μg/ml) diluted in 25 mM Briton-Robinson buffer (pH 5 and 6) and mixed with 2.5 μl of SYPRO Orange was used as a positive control. A curve experiment for DNA with the elimination of the initial denaturation step at 95°C was used. The melt curve temperature range was set from 20°C to 95°C in increments of 0.5°C for 5 s. Each experimental condition was prepared in triplicate. Thermal shift analysis or differential scanning fluorimetry (DSF) of Geo6 was performed in CFX Touch Real TIME PCR System (Bio-Rad) using CFX Manager (Bio-Rad) software.

## 3. Results and discussion

### 3.1. Identification of biosynthetic gene cluster of geobacillin 6

**Figure 1:**
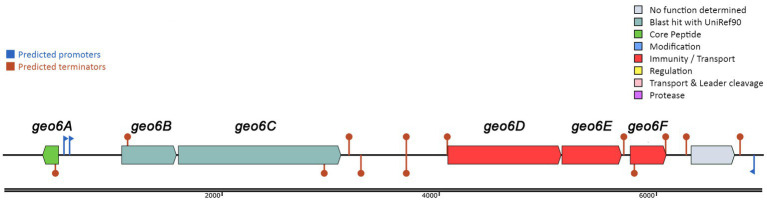
Biosynthetic gene cluster of bacteriocin Geo6 identified using BAGEL4 web tool. Represented genes *geo6ABCDEF* in DNA contig (GenBank: LAKX01000053.1) were identified in the genome of *P. thermoglucosidasius* DSM 2542 (GenBank: LAKX00000000).

**Figure 2:**
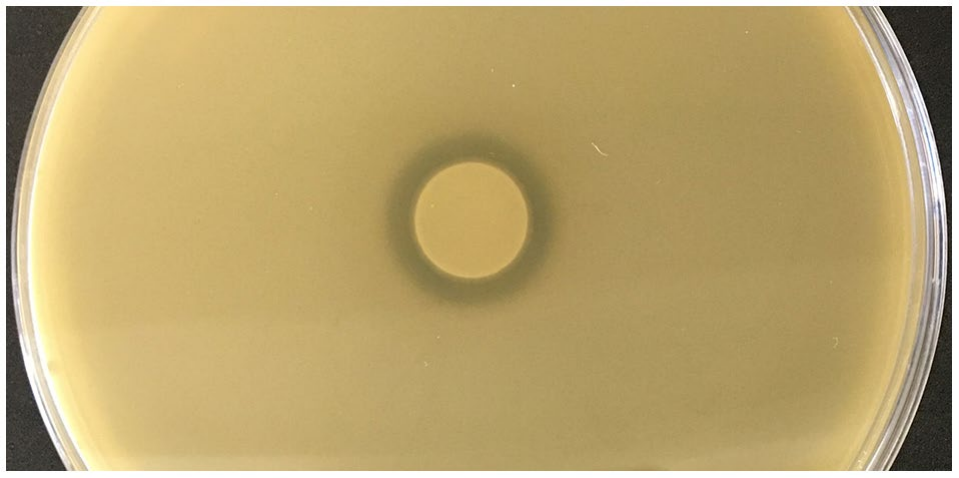
Assessment of antimicrobial activity of *P. thermoglucosidasius* DSM 2542. Indicator strain inoculated into the agar plate - *Geobacillus kaustophilus* HTA 426, bacterial suspension spotted into the center of the plate - *P. thermoglucosidasius* DSM 2542.

To reveal new antimicrobial peptides encoded in thermophilic bacteria, we screened a series of NCBI-available genomes from various *Geobacillus* and *Parageobacillus* spp. bacteria stored in the microorganism collection of the Department of Microbiology and Biotechnology at Vilnius University’s Life Sciences Center. Genome analysis using the BAGEL4 web tool revealed that the genomic DNA of the thermophilic bacteria *P. thermoglucosidasius* DSM 2542 encoded a gene cluster of 5,709 bp in length (Figure 1) and presumably is responsible for the biosynthesis of a novel bacteriocin. This strain was confirmed to produce antimicrobial substances using a spot on a lawn assay (Figure 2). The gene cluster contained 6 genes, which were named here *geo6ABCDEF*, could be related to the biosynthesis of the novel bacteriocin, that we have named geobacillin 6 (Geo6).

**Figure 3:**
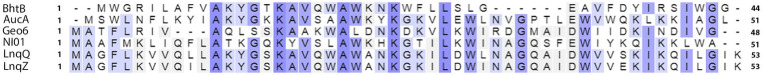
Sequence alignment of leaderless bacteriocins. Alignment of peptides: BhtB (UniProt: Q3YB73), AucA (UniProt: Q8GPI4), NI01 (UniProt: H9BG66), LnqQ (UniProt: A4UVR2), LnqZ (UniProt: A7M6Q0), Geo6 (GeneBank: KJX67188.1), were performed using the UniProt database.

**Figure 4:**
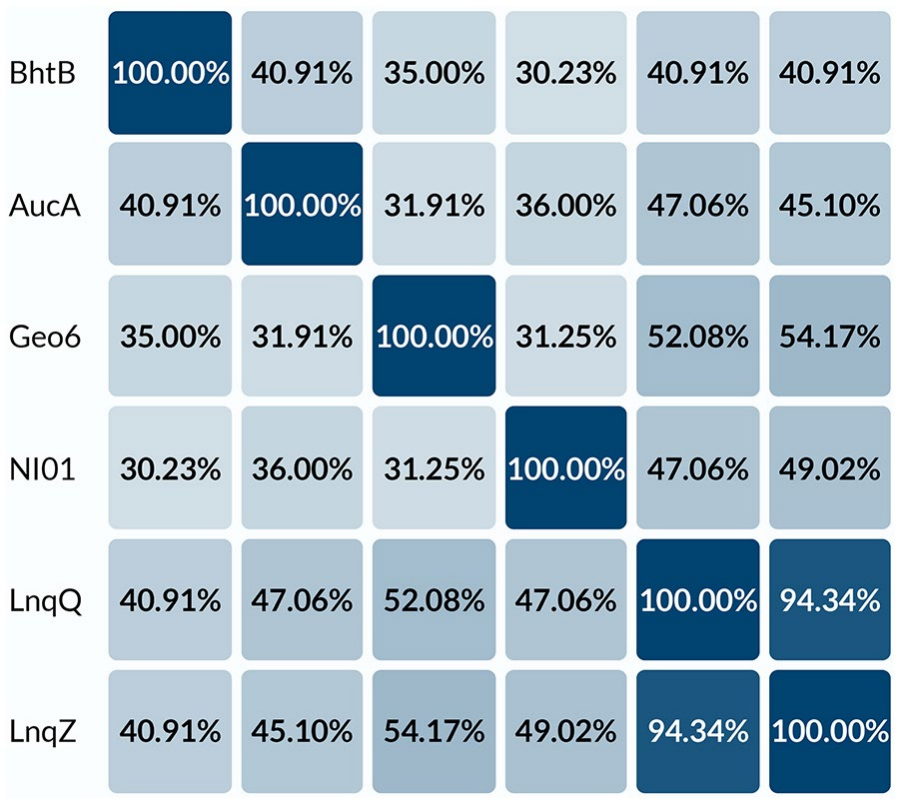
Percent identity matrix of leaderless bacteriocins. Alignment of peptides: BhtB (UniProt: Q3YB73), AucA (UniProt: Q8GPI4), NI01 (UniProt: H9BG66), LnqQ (UniProt: A4UVR2), LnqZ (UniProt: A7M6Q0), Geo6 (GeneBank: KJX67188.1), were performed using UniProt database.

BLAST analysis and alignment of the *geo6A* gene product (Figures 3, 4) revealed that the encoded 48 amino acid length peptide, Geo6, shares sequence similarity with a group of leaderless bacteriocins: lacticins Q and Z, aureocin A54, epidermicin NI01, and BhtB (Perez et al., 2018). The highest sequence similarity was observed with lacticin Q (52%) and lacticin Z (54%).

Further analysis of the gene cluster observed that the *geo6D, geo6E,* and *geo6F* genes encode proteins with 78, 78, and 84% sequence similarity, respectively, to proteins annotated as ABC transporters. While the *geo6B* and *geo6C* genes encode proteins with 51 and 34% sequence similarity, respectively, to proteins annotated as Pleckstrin homology (PH) domain-containing proteins. Additionally, they exhibit 46 and 29% similarity, respectively, to proteins annotated as transmembrane YdbS and YdbT proteins, respectively.

The PH domains are small modular domains, which occur in a large variety of signaling proteins and serve as simple targeting domains that bind lipids in eukaryotic cells. However, these proteins are also present in bacteria (Cozier et al., 2004; Xu et al., 2010). Based on Butcher and Helmann (2006) research, the YdbST proteins can be responsible for resistance to antimicrobial compound(s) produced by *Bacillus amyloliquefaciens*. A recent study suggests that proteins containing PH domains and proteins similar to YbdST constitute transporters dedicated to leaderless bacteriocins (Pérez-Ramos et al., 2021). In addition, studies of gene clusters coding for bacteriocin lacticin Q (Iwatani et al., 2013) and aureocin A54 (Nascimento et al., 2012) showed that their biosynthetic loci encode proteins similar to ABC transporters. The secretion of the lacticin Q in *L. lactis* QU5 is strictly controlled by the presence of LnqBCDEF, which confers the secretion and self-immunity of lacticin Q. It was revealed that the LnqEF proteins are the minimal unit required for sufficient immunity. Whereas LnqBCD, showing sequence similarity to ABC transporters, are considered accessory proteins, which support the activity of the LnqEF (Iwatani et al., 2012, 2013).

However, studies have demonstrated that the production of leaderless bacteriocins can occur independently of the biosynthesis machinery. It has been shown that the synthesis of active bacteriocin is possible with the production of only the precursor peptide (Netz et al., 2002b).

Based on previous studies and analysis of the *geo6ABCDEF* genes, it can be suggested that these genes are responsible for the biosynthesis of Geo6 (Table 1). The *geo6A* gene encodes the bacteriocin precursor peptide Geo6, while the *geo6BCDEF* genes are dedicated to transport and immunity, as observed in other biosynthetic gene clusters of leaderless bacteriocins. Some gene clusters of leaderless bacteriocins also contain genes responsible for biosynthesis regulation (Perez et al., 2018). However, in the identified gene cluster, which was encoded in a relatively short DNA contig (6,966 bp, GenBank: LAKX01000053.1), only one gene outside of *geo6ABCDEF* was found. No other genes associated with bacteriocin biosynthesis or regulation were identified.

**Table 1:** Annotation of identified gene cluster.

Gene name	*geo6A*	*geo6B*	*geo6C*	*geo6D*	*geo6E*	*geo6F*
Annotation	Lacticin Q-like, aureocin A53-like peptide	PH domain-containing protein, transmembrane protein YdbS	PH domain-containing protein, transmembrane protein YdbT	ABC transporter domain-containing protein, bacitracin transporter	ABC transporter, permease YhcI	ABC transporter, permease YhcI
Proposed function	Bacteriocin precursor	Transport/immunity	Transport/immunity	Transport/immunity	Transport/immunity	Transport/immunity

### 3.2. Biosynthesis and purification of Geo6

**Figure 5:**
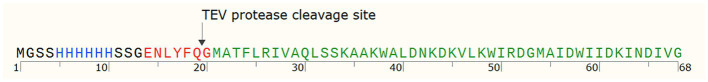
His-TEV-Geo6 peptide sequence. Blue depicts His-tag sequence for Ni^+2^ affinity chromatography purification, red depicts the TEV protease recognition site, green depicts Geo6 bacteriocin sequence.

**Figure 6:**
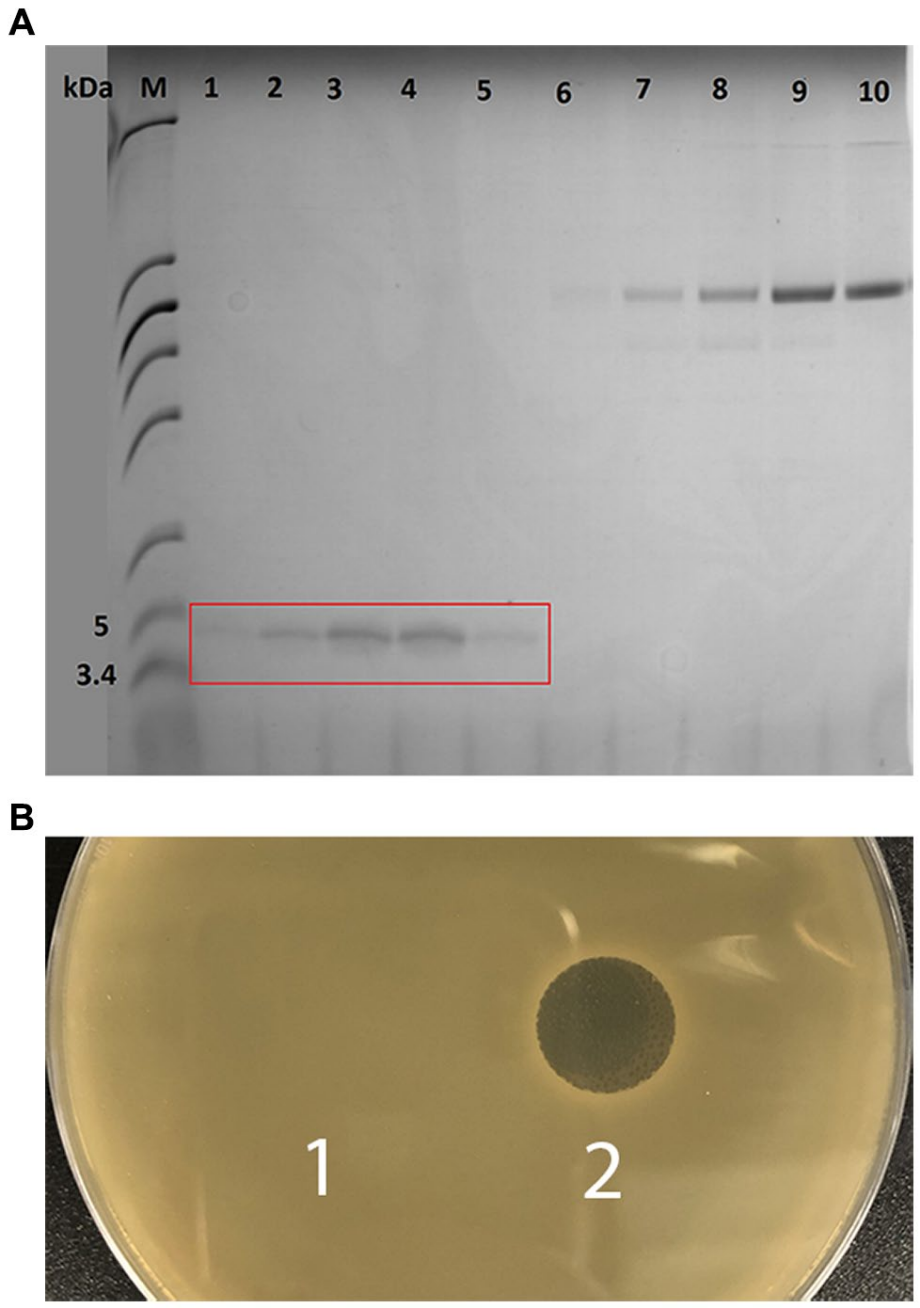
Analysis of purified recombinant Geo6 peptide. **(A)** Tricine-SDS-PAGE analysis of Geo6 after ion-exchange chromatography. Lane M represents molecular weight marker PageRuler Unstained Low Range Protein Ladder (Thermo Scientific). Lanes 1–10 represent elution fractions, with lanes 1–5 corresponding to eluted Geo6 and lanes 6–10 representing separated impurities and other proteins. **(B)** Spot-on-lawn assay. The indicator strain used in the antimicrobial assay was the thermophilic bacteria *G. kaustophilus* HTA 426, which was incubated overnight at 55°C. 1 – elution buffer (control), 2 – purified Geo6 peptide.

Further, gene *geo6A* was heterologously expressed in *E. coli* for biosynthesis and purification of recombinant bacteriocin. For this purpose, we used a synthetic gene with optimized codons for *E. coli* bacteria. In addition, the gene was designed to encode His6-tag and TEV peptidase recognition site in the N-terminus of the peptide (Figure 5). The new gene of bacteriocin precursor, *His-TEV-geo6A*, was cloned into pET-15b vector downstream T7 promoter and induced for expression in *E. coli* BL21 (DE3) cells. The synthesized peptide His-TEV-Geo6 was purified from soluble cell lysate fraction using Ni^+2^ affinity chromatography, desalted by gel chromatography, then treated with TEV protease to cleave His-tag sequence and finally purified to homogeneity by cation exchange chromatography (Figure 6A). The recombinant bacteriocin Geo6 had additional Gly upstream of the Met at the N-terminus of the peptide.

### 3.3. Determination of antibacterial activity spectrum and minimal inhibitory concentration of recombinant geobacillin 6

Purified Geo6 peptide was evaluated for the antibacterial activity using spot on a lawn assay against 36 bacteria and 4 yeast strains, including Gram-positive, Gram-negative, and pathogenic strains (Table 2). Results demonstrated that bacteriocin was active against 12 out of 19 thermophilic bacteria strains tested, including *Aeribacillus pallidus* DSM 3670^T^, *Anoxybacillus tepidamans* DSM 16325^T^, *Parageobacillus toebii* DSM 14590^T,^ and various *Geobacillus* spp. bacteria strains (Figure 6B). Additionally, we observed small antibacterial activity against one mesophilic bacterium, *Bacillus zanthoxyli* CH07. However, the yeasts *Saccharomyces cerevisiae* or *Candida* spp., as well as the mesophilic bacteria *Bacillus* spp., *Staphylococcus* spp., *Pseudomonas aeruginosa*, *Enterococcus faecalis*, *Salmonella enterica*, *Escherichia coli*, *Acinetobacter baumannii*, and *Stenotrophomonas maltophilia* strains used in the assay were not susceptible to the bacteriocin.

**Table 2:** Antibacterial activity spectrum and MIC values of Geo6.

Microorganism name	Strain name	Growth temperature	Growth inhibition	MIC
*Aeribacillus pallidus*	DSM 3670^T^	55°C	+++	NA
*Anoxybacillus tepidamans*	DSM 16315^T^	55°C	−	NA
*Anoxybacillus tepidamans*	DSM 16325^T^	55°C	+	NA
*Geobacillus stearothermophilus*	DSM 22^T^	55°C	+	NA
*Geobacillus stearothermophilus*	DSM 13240	55°C	+	NA
*Geobacillus lituanicus*	DSM 15325^T^	55°C	+++	585 nM
*Geobacillus gargensis*	DSM 15378^T^	55°C	+	NA
*Geobacillus caldoxylosilyticus*	DSMZ 12833	55°C	−	NA
*Geobacillus debilis*	DSM 16016^T^	55°C	++	NA
*Geobacillus jurassicus*	DSM 15726^T^	55°C	+	NA
*Geobacillus kaustophilus*	HTA 426	55°C	+++	293 nM
*Geobacillus subterraneus*	DSM 13552^T^	55°C	−	NA
*Geobacillus thermocatenulatus*	DSM 730	55°C	−	NA
*Geobacillus thermodenitrificans*	DSM 465^T^	55°C	++	2.34 μM
*Geobacillus thermoleovorans*	DSM 5366^T^	55°C	+++	585 nM
*Geobacillus uzenensis*	DSM 13551^T^	55°C	−	NA
*Parageobacillus genomospecies 1*	NUB 36187	55°C	−	NA
*Parageobacillus thermoglucosidasius*	DSM 2542^T^	55°C	−	NA
*Parageobacillus toebii*	DSM 14590^T^	55°C	+	4,45 μM
*Bacillus spizizenii*	ATCC 6633	37°C	−	NA
*Bacillus subtilis*	168	37°C	−	NA
*Bacillus velezensis*	CH02	37°C	−	NA
*Bacillus zanthoxyli*	CH07	37°C	+	NA
*Pseudomonas aeruginosa*	ATCC 27853 (P)	37°C	−	NA
*Staphylococcus epidermidis*	ATCC 12228 (P)	37°C	−	NA
*Staphylococcus aureus subsp. aureus*	ATCC 25923 (P)	37°C	−	NA
*Staphylococcus saprophyticus*	AG1	37°C	−	NA
*Enterococcus faecalis*	DSMZ 2570 (P)	30°C	−	NA
*Salmonella enterica serovar Typhimurium*	LK (P)	30°C	−	NA
*Saccharomyces cerevisiae*	α′1	30°C	−	NA
*Candida lusitaniae*	CL18 (P)	30°C	−	NA
*Candida guilliermondii*	EL (P)	30°C	−	NA
*Candida albicans*	ATCC 14053 (P)	30°C	−	NA
*Acinetobacter baumannii*	Ab171 (P)	37°C	−	NA
*Acinetobacter baumannii*	Ab169 (P)	37°C	−	NA
*Acinetobacter baumannii*	Ab141 (P)	37°C	−	NA
*Stenotrophomonas maltophilia*	SM21 (P)	37°C	−	NA
*Stenotrophomonas maltophilia*	SM3 (P)	37°C	−	NA
*Stenotrophomonas maltophilia*	D53 (P)	37°C	−	NA
*Escherichia coli*	BL21 (DE3)	37°C	−	NA

P refers to pathogenic strain.

In addition, the MIC values of Geo6 were evaluated against some susceptible thermophilic bacteria strains (Table 2). It was demonstrated that the MIC value for *G. thermodenitrificans* DSM 465^T^ was 2.34 μM, for *G. lituanicus* DSM 15325^T^ – 585 nM, for *G. thermoleovorans* DSM 5366^T^ – 585 nM, for *G. kaustophilus* HTA 426–293 nM, and for *P. toebii* DSM 14590^T^ it was 4.45 μM.

For comparison, previous studies have shown that pallidocin, a glycocin-type bacteriocin produced by thermophilic bacteria *A. pallidus* strain 8, was active in the pM range against other thermophiles, and a 2.4 pM concentration of pallidocin inhibited the growth of thermophilic bacterium *P. genomospecies* 1 strain NUB36187 (Kaunietis et al., 2019). Other leaderless bacteriocins are active in the μM and nM ranges (Fujita et al., 2007; Iwatani et al., 2007) and have a broad spectrum of activity. Studies showed that lacticin Q was active against a variety of tested Gram-positive bacteria strains, including *Bacillus* spp., *Lactobacillus* spp., *Enterococcus* spp., *Pediococcus* spp., *Lactococcus* spp., *Streptococcus* spp., and some other species. The highest activity was detected against *B. coagulans* JCM 2257^T^ (MIC – 1.6 nM), *L. sakei* subsp. *sakei* JCM 1157^T^ (MIC – 3.2 nM), *P. dextrinicus* JCM 5887^T^ (MIC – 7.3 nM). No activity was detected against the Gram-positive bacteria *Escherichia coli* JM109 (Iwatani et al., 2007). Bacteriocin aureocin A54 had a narrower antibacterial activity spectrum, it was active against some tested Gram-positive indicator bacteria strains, including *Brochothrix* sp., *Carnobacterium* spp., *Lactococcus* spp., and *Staphylococcus* spp. The lowest MIC values were detected against *B. campestris* ATCC 43754–2 μM, and *L. lactis* ATCC 19257–250 nM (Acedo et al., 2016).

Our results show that Geo6, as well as other leaderless bacteriocins lacticin Q or aureocin A54, are active in nM concentrations, in contrast to the bacteriocin pallidocin from another thermophilic bacterium, which can kill bacteria in pM concentrations. However, Geo6, similar to pallidocin, is active against Gram-positive and mostly against closely related thermophilic bacteria, including *Geobacillus* spp., *Aeribacillus* sp., *Anoxybacillus* sp., and *Parageobacillus* sp. strains. Other leaderless bacteriocins, such as lacticin Q and aureocin A54, inhibit Gram-positive bacteria as well. However, they have a wider antibacterial spectrum and kill more than just closely related bacteria.

### 3.4. Determination of mode of action

**Figure 7:**
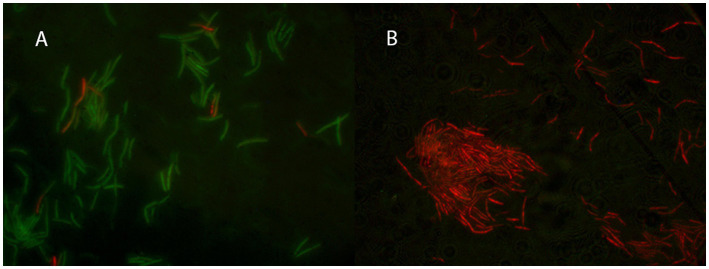
Fluorescent microscopy of thermophilic bacteria *G. kaustophilus* HTA 426^T^ cells. In **(A)** the control cells not treated with Geo6, while in **(B)** bacteria cells were treated with Geo6 for 30 min. The cells were stained using fluorescent dyes SYTO9 and PI. SYTO9 is capable of permeating the cell membranes and binding to the DNA of both live and dead cells. PI can only enter cells with damaged membranes and binds to the DNA. In **(A)** most of the cells exhibited green fluorescence, indicating that they were alive and healthy, with only a few cells showing red fluorescence due to membrane damage. In **(B)** all the cells exhibited red fluorescence, indicating that they were dead, as their membranes were no longer intact and the PI dye was able to bind to the DNA.

Cells of *G. kaustophilus* HTA 426^T^ were treated with Geo6, and membrane integrity was assayed using the fluorescent dyes SYTO9 and PI (Figure 7). The experiment showed that bacteria cells treated with the bacteriocin emitted red fluorescence, since propidium iodide can enter only cells with damaged membranes. In contrast, control cells not exposed to Geo6 emitted green fluorescence, indicating an intact membrane. These results suggest that Geo6 destabilizes the membrane of indicator cells. This can be supported by the fact that the peptide has a high charge, its theoretical isoelectric point (pI) value is 9.3. Cationic peptides tend to be attracted to an anionic cell membrane. Moreover, our suggestion that Geo6 targets the cell membrane is in agreement with a recent study on leaderless bacteriocins, epidermicin NI01 (*Staphylococcus epidermidis*) and aureocin A53, which revealed the unique mode of action of these peptides. It comprises several distinctive methods of membrane disruption, and it was found that a four-helix bundle structure is necessary to complete the mechanism of membrane disruption (Hammond et al., 2020). Research on aureocin A53 and lacticin Q showed permeabilization of the bacterial cell membrane, followed by membrane potential disruption, efflux of various vital compounds, and termination of macromolecular synthesis (Netz et al., 2002a; Fujita et al., 2007). It was also shown that lacticin Q forms at least 4.6 nm pores (Yoneyama et al., 2009).

### 3.5. Geo6 stability at different temperatures and pH ranges

The purified recombinant Geo6 was exposed to different temperatures, and the thermal stability of the bacteriocin was evaluated after treatment by using a spot-on-lawn assay. The results showed that after treatment at 60°C, 70°C, and even 100°C for 2 h, the peptide retained 100% of its antibacterial activity. Furthermore, incubation of the peptide for up to 6 h at 95°C did not abolish its activity, and after incubation at room temperature for 24 h, the peptide remained 100% active. Additionally, Geo6 stability was evaluated in different pH ranges. Incubation in the pH range of 3–11 showed that Geo6 retained 100% of its activity in acidic and alkaline environments. These results are consistent with observations from experiments with other leaderless bacteriocins, such as lacticin Q or Bht-B, which remained active after treatment at high temperatures and in a wide pH range (Fujita et al., 2007).

### 3.6. Geo6 structure prediction

**Figure 8:**
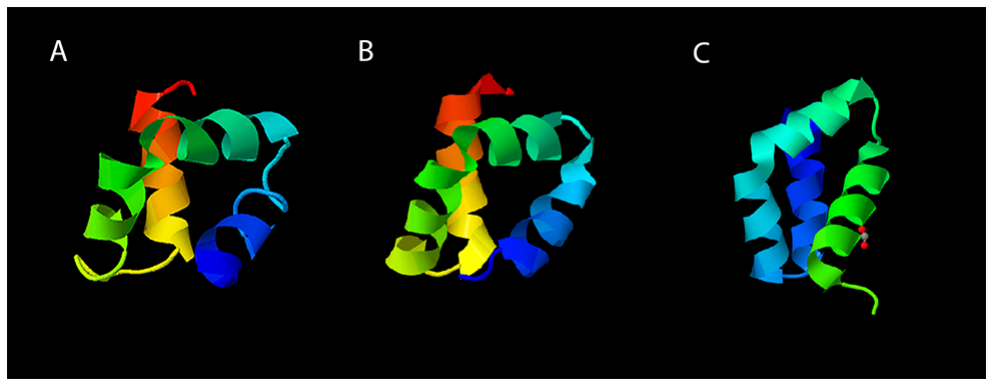
Three-dimensional structures of leaderless bacteriocins. The structure of Geo6 **(A)** was predicted using I-TASSER web tool. Experimentally determined 3D structures of AucA **(B)** and LnqQ **(C)** were derived from the RCSB PDB archive. The PDB numbers for AucA and LnqQ are 2N8O and 7P5R, respectively. In the visualizations, different colors are assigned to the different chains within the peptide structure. This color scheme distinguishes and visualizes the individual components of the peptide. The N-terminus is represented in blue, followed by green, yellow, orange, and finally red for the C-terminus.

**Figure 9:**
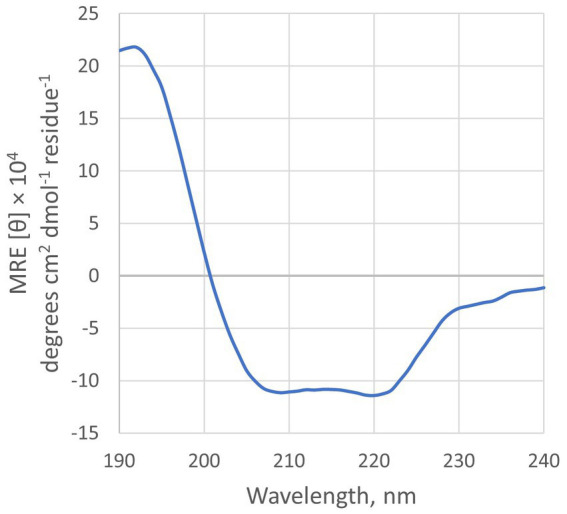
CD spectra of Geo6. Conditions: 10 μM of peptide in 10 mM sodium phosphate buffer (pH 7.4), 25°C.

Analysis of the Geo6 peptide sequence using the SignalP bioinformatics tool confirmed that it has no signal or leader peptide, consistent with other leaderless bacteriocins. Three-dimensional structure prediction using I-TASSER suggested that Geo6 is predominantly formed from α-helixes. Furthermore, structure assembly simulation showed that Geo6 forms four distinct helixes, which fold into a compact, globular assembly (Figure 8), similar to other leaderless bacteriocins such as epidermicin NI01 (PDB: 6SIF), aureocin A54 (PDB: 2N8O) and lacticin Q (PDB: 2N8P). Previous studies using structure prediction tools suggested a similar assembly for other leaderless bacteriocins such as lacticin Z, weissellicins Y and M, and enterocins L50A and L50B (Acedo et al., 2016). The secondary structure of Geo6 was evaluated using circular dichroism (CD) spectroscopy (Figure 9). The CD spectra revealed the presence of α-helical structure of the peptide in an aqueous solution. These findings are consistent with the predictions made by I-TASSER and are in agreement with previous studies on epidermicin NI01 and aureocin A54 (Hammond et al., 2020). Computational analysis of the spectra further estimated that α-helices may make up 75% of the peptide. It is well-known that the presence of α-helices and their enhanced conformational stability contribute significantly to the thermal stability of proteins (Yakimov et al., 2016). This observation provides a plausible explanation for the remarkable stability of Geo6 at high temperatures.

### 3.7. Geo6 analysis using thermal shift assay

A recent study using the CD spectroscopy method confirmed the formation of helical structures in epidermicin NI01 and aureocin A53 in aqueous buffers. It also revealed that epidermicin NI01 undergoes denaturation at 60°C and exhibits a reversible two-state conversion between helical and unfolded forms (Hammond et al., 2020). Due to these interesting findings, we decided to employ a thermal shift assay, also known as differential scanning fluorimetry (DSF), to analyze the thermal denaturation of Geo6 under different pH ranges and temperatures. Additionally, to the best of our knowledge, this method has never been employed to study the stability and structural changes of bacteriocins.

The fluorescent dye (SYPRO Orange) used in this assay is highly fluorescent in non-polar environments, such as hydrophobic sites on unfolded proteins, compared to an aqueous solution where the fluorescence is quenched. By monitoring the increase in fluorescence, we can observe the binding of the dye to the hydrophobic amino acids of the unfolded peptide, which are normally hidden within the helical structure. Additionally, the DSF method enables the use of small sample volumes and low concentrations (Niesen et al., 2007).

**Figure 10:**
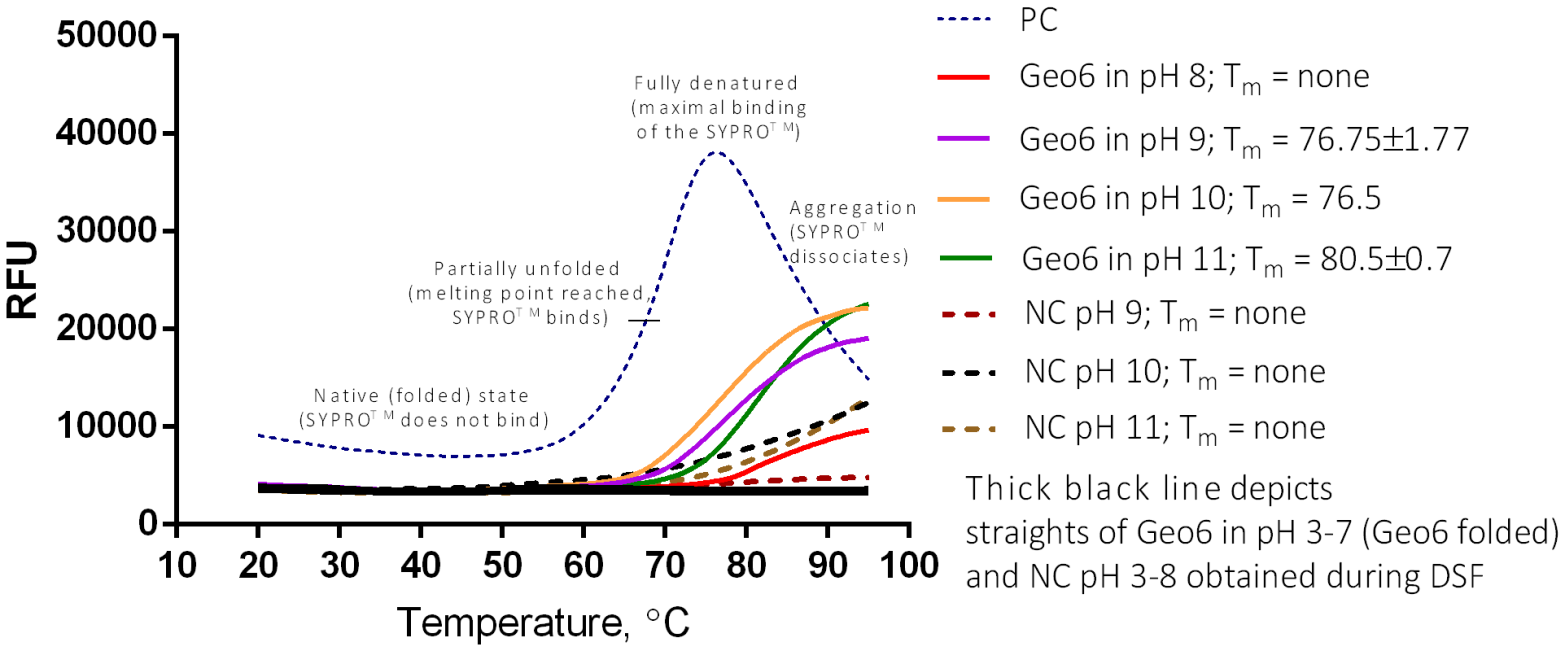
DSF or thermal shift of Geo6 bacteriocin. All Geo6 samples were suspended in 25 mM Briton-Robinson buffer with different pH values (pH 3–11) and with SYPRO Orange dye. PC – positive control of 100 μg/ml lysozyme solution in 25 mM Briton-Robinson buffer, pH 6, and with SYPRO Orange dye. NC – negative controls in 25 mM Briton-Robinson buffer solutions (pH 3–11) with SYPRO Orange dye. Samples with Geo6 (pH 3–7) and NCs (pH 3–8) depicted in black straight lines indicate no fluorescence change. NCs in pH 9–11 depicted in colored dotted lines indicate slight fluorescence change. Samples with Geo6 in pH 8–11 depicted in colored straight lines indicate moderate fluorescence change. PC displays a typical sigmoidal line of unfolded protein, demonstrating significant fluorescence change. RFU – relative fluorescence units.

The analysis showed that Geo6 remained in its unfolded state over a range of pH 3–7 during the increase of temperatures from 20°C to 95°C, and the formation of typical melt curves did not occur during the DSF assay (Figure 10). Such results imply that Geo6 remained extremely stable under the DSF conditions and stand in line with the results obtained in the spot on a lawn assay. Partial unfolding or thermal denaturation of Geo6 was detected in the range of pH 9–11, implying that in these conditions the peptide starts to denature (melting temperatures (T_m_) of 76.5–80.5°C were determined). However, the observed melting curves did not indicate full denaturation or aggregation. A slight increase in relative fluorescence units (RFU) was also detected at pH 8, and the appropriate negative controls within the pH range of 9–11 also exhibited increased RFU. As a result, the findings of the DSF assay for Geo6 within the pH range of 8–11 are inconclusive and represent a major limitation of this study in accurately determining the stability of Geo6 within this pH range. Given that Geo6 has a pI value of 9.3, it can become negatively charged at pH levels higher than its pI, potentially leading to the destabilization of the bacteriocin. Despite the inconclusive results at pH 8–11, the denaturation or unfolding of Geo6 within the pH range 3–7 was not observed, which contradicts the findings of the previous study on epidermicin NI01 and aureocin A53 using CD spectroscopy analysis (Hammond et al., 2020).

## 4. Conclusion

To date, at least 33 antimicrobial peptides falling into the leaderless bacteriocin subclass have been identified and characterized. Their molecular weight varies between 2.8–6.0 kDa and they are produced mainly by mesophilic *Enterococcus*, *Weissella*, *Staphylococcus*, *Lactococcus*, *Bacillus,* and *Streptococcus* spp. bacteria (Perez et al., 2018). This study demonstrates that thermophilic strain *P. thermoglucosidasius* DSM 2542 encodes a novel cationic leaderless bacteriocin, Geo6, with a molecular weight of 5.5 kDa. Three-dimensional structure prediction and CD spectroscopy determined that it resembles structures of other leaderless bacteriocins, comprising the helical structure. The bacteriocin was heterologously synthesized in *E. coli* and purified using chromatographic methods.

Our findings demonstrated that Geo6 has extreme stability in high temperatures and a wide pH range. Geo6 is active in μM and nM concentrations, as other leaderless bacteriocins as lacticins Q/Z work in the nM range too (Fujita et al., 2007; Iwatani et al., 2007). Moreover, only one *Bacillus* sp. strain and some thermophilic *Geobacillus* spp., *Parageobacillus* sp. or *Aeribacillus* sp. strains demonstrated susceptibility to Geo6 which shows bacteriocin has a narrow antibacterial activity spectrum and is active mostly against thermophilic bacteria. The determined mode of action showed that Geo6 works through cytoplasmic membrane permeabilization, in a similar way to other leaderless bacteriocins (Hammond et al., 2020).

Considering Geo6 stability in harsh environments and its activity against thermophilic bacteria, it could be applied in the food industry and in biotechnological processes where contamination with thermophilic bacteria is undesirable. Furthermore, the identification and characterization of novel bacteriocins like Geo6 contribute to our understanding of the diversity of bacteriocins and expand our knowledge about antimicrobial peptides. This knowledge, in turn, can facilitate the adoption of antimicrobial peptides in diverse biotechnological sectors.

Leaderless bacteriocins, such as Geo6, possess significant potential in various applications due to their simple structure compared to other bacteriocins. They can be fused with signal peptides of other proteins, in this way production at high yields and secretion in other microorganisms or eukaryotic cells could be easily achieved. In addition, the determination of bacteriocin structure in genes allows their easy modifications and improvements.

## Data availability statement

The original contributions presented in the study are included in the article/Supplementary material, further inquiries can be directed to the corresponding author.

## Author contributions

AKa: conceptualization, methodology, investigation, supervision, funding acquisition, and writing – original draft. AKo, AP, EB, and AGe: investigation. AGr: investigation, and writing – review and editing. LK: funding acquisition. All authors contributed to the article and approved the submitted version.

## Funding

This project has received funding from European Social Fund (project no 09.3.3-LMT-K-712-22-0095) under a grant agreement with the Research Council of Lithuania (LMTLT).

## Conflict of interest

The authors declare that the research was conducted in the absence of any commercial or financial relationships that could be construed as a potential conflict of interest.

## Publisher’s note

All claims expressed in this article are solely those of the authors and do not necessarily represent those of their affiliated organizations, or those of the publisher, the editors and the reviewers. Any product that may be evaluated in this article, or claim that may be made by its manufacturer, is not guaranteed or endorsed by the publisher.

## Footnotes

^1^https://www.ncbi.nlm.nih.gov/genome

^2^http://bagel4.molgenrug.nl

^3^https://services.healthtech.dtu.dk/service.php?SignalP-5.0

^4^https://blast.ncbi.nlm.nih.gov/Blast.cgi

^5^https://www.uniprot.org/blast

^6^https://www.uniprot.org/align

^7^https://zhanggroup.org/I-TASSER

^8^http://dichroweb.cryst.bbk.ac.uk/html/home.shtml
