# Supplementary material for: Novel leaderless bacteriocin geobacillin 6 from thermophilic bacterium Parageobacillus thermoglucosidasius
Source: Front Microbiol. 2023 Jun 15;14:1207367. doi: 10.3389/fmicb.2023.1207367 (PMC10311245; doi:10.3389/fmicb.2023.1207367)
Supplement: Supplementary file 1 [file Data_Sheet_1.docx]

Supplementary Material

Novel leaderless bacteriocin geobacillin 6 from thermophilic bacterium *Parageobacillus thermoglucosidasius*

Ana Koniuchovaitė^1^, Akvilė Petkevičiūtė^1^, Emilija Bernotaitė^1^, Alisa Gricajeva^1^, Audrius Gegeckas^1^, Lilija Kalėdienė^1^, Arnoldas Kaunietis^1*^

^1^ Department of Microbiology and Biotechnology, Institute of Biosciences, Life Sciences Center, Vilnius University, Saulėtekio ave. 7, Vilnius LT-10257, Lithuania.

*** Correspondence:** Arnoldas Kaunietis, [arnoldas.kaunietis@gmc.vu.lt](mailto:arnoldas.kaunietis@gmc.vu.lt).

The designed DNA sequence encoding His-TEV-Geo6 peptide (underlined) containing NcoI and BamHI restriction sites (in bold):

>His-TEV-Geo6

TGTTTAACTTTAAGAAGGAGATATA**CCATGG**GCAGCAGCCATCACCATCATCATCATAGCAGCGGTGAAAACCTGTATTTTCAAGGTATGGCAACCTTTCTGCGTATTGTTGCACAGCTGAGCAGCAAAGCAGCAAAATGGGCATTAGATAACAAAGACAAAGTGCTGAAATGGATTCGTGATGGTATGGCCATTGATTGGATCATCGATAAAATCAACGACATCGTCGGCTAA**GGATCC**GGCTGCTAACAAAGCCCGAAAGGAA
