# Supplementary material for: Novel leaderless bacteriocin geobacillin 6 from thermophilic bacterium Parageobacillus thermoglucosidasius
Source: Front Microbiol. 2023 Jun 15;14:1207367. doi: 10.3389/fmicb.2023.1207367 (PMC10311245; doi:10.3389/fmicb.2023.1207367)
Supplement: Supplementary file 2 [file Table_1.docx]

Supplementary Material

Novel leaderless bacteriocin geobacillin 6 from thermophilic bacterium *Parageobacillus thermoglucosidasius*

Ana Koniuchovaitė^1^, Akvilė Petkevičiūtė^1^, Emilija Bernotaitė^1^, Alisa Gricajeva^1^, Audrius Gegeckas^1^, Lilija Kalėdienė^1^, Arnoldas Kaunietis^1*^

^1^ Department of Microbiology and Biotechnology, Institute of Biosciences, Life Sciences Center, Vilnius University, Saulėtekio ave. 7, Vilnius LT-10257, Lithuania.

*** Correspondence:** Arnoldas Kaunietis, [arnoldas.kaunietis@gmc.vu.lt](mailto:arnoldas.kaunietis@gmc.vu.lt).

**Table S1**. List of the strains and growth conditions used in antimicrobial assay. DSMZ - Leibniz Institute DSMZ-German Collection of Microorganisms and Cell Cultures; ATCC - American Type Culture Collection; JCM - Japan Collection of Microorganisms.

| **Microorganism name** | **Strain name** | **Source, reference** | **Medium for a spot on a lawn assay** | **Medium for a MIC assay** | **Growth temperature** |
| --- | --- | --- | --- | --- | --- |
| *Aeribacillus pallidus* | DSM 3670^T^ | DSMZ | CASO/NB | NA | 55°C |
| *Anoxybacillus tepidamans* | DSM 16315^T^ | DSMZ | CASO/NB | NA | 55°C |
| *Anoxybacillus tepidamans* | DSM 16325^T^ | DSMZ | CASO/NB | NA | 55°C |
| *Geobacillus stearothermophilus* | DSM 22^T^ | DSMZ | CASO/NB | NA | 55°C |
| *Geobacillus stearothermophilus* | DSM 13240 | DSMZ | CASO/NB | NA | 55°C |
| *Geobacillus lituanicus* | DSM 15325^T^ | Kuisiene et al., 2004 | CASO/NB | BHI | 55°C |
| *Geobacillus gargensis* | DSM 15378^T^ | DSMZ | CASO/NB | NA | 55°C |
| *Geobacillus caldoxylosilyticus* | DSMZ 12833 | DSMZ | CASO/NB | NA | 55°C |
| *Geobacillus debilis* | DSM 16016^T^ | DSMZ | CASO/NB | NA | 55°C |
| *Geobacillus jurassicus* | DSM 15726^T^ | DSMZ | CASO/NB | NA | 55°C |
| *Geobacillus kaustophilus* | HTA 426 (JCM 12893) | JCM | CASO/NB | BHI | 55°C |
| *Geobacillus subterraneus* | DSM 13552^T^ | DSMZ | CASO/NB | NA | 55°C |
| *Geobacillus thermocatenulatus* | DSM 730 | DSMZ | CASO/NB | NA | 55°C |
| *Geobacillus thermodenitrificans* | DSM 465^T^ | DSMZ | CASO/NB | BHI | 55°C |
| *Geobacillus thermoleovorans* | DSM 5366^T^ | DSMZ | CASO/NB | BHI | 55°C |
| *Geobacillus uzenensis* | DSM 13551^T^ | DSMZ | CASO/NB | NA | 55°C |
| *Parageobacillus genomospecies 1* | NUB 36187 | BGSC | CASO/NB | NA | 55°C |
| *Parageobacillus thermoglucosidasius* | DSM 2542^T^ | DSMZ | CASO/NB | NA | 55°C |
| *Parageobacillus toebii* | DSM 14590^T^ | DSMZ | CASO | CASO | 55°C |
| *Bacillus subtilis* | ATCC 6633 | ATCC | LB | NA | 37°C |
| *Bacillus subtilis* | 168 (DSM 23778) | DSMZ | LB | NA | 37°C |
| *Bacillus velezensis* | CH02 | Lab strain | LB | NA | 37°C |
| *Bacillus zanthoxyli* | CH07 | Lab strain | LB | NA | 37°C |
| *Pseudomonas aeruginosa* | ATCC 27853 | ATCC, pathogenic | LB | NA | 37°C |
| *Staphylococcus epidermidis* | ATCC 12228 | ATCC, pathogenic | LB | NA | 37°C |
| *Staphylococcus aureus* | ATCC 25923 | ATCC, pathogenic | LB | NA | 37°C |
| *Staphylococcus saprophyticus* | AG1 | Gricajeva et al., 2019 | LB | NA | 37°C |
| *Enterococcus faecalis* | DSM 2570 | DSMZ, pathogenic | LB | NA | 30°C |
| *Salmonella enterica serovar Typhimurium* | LK | Lab strain, pathogenic | LB | NA | 30°C |
| *Saccharomyces cerevisiae* | α′1 | Lab strain | LB | NA | 30°C |
| *Candida lusitaniae* | CL18 | Zinkeviciene et al., 2011, pathogenic | YPD | NA | 30°C |
| *Candida guilliermondii* | EL | Lab strain, pathogenic | YPD | NA | 30°C |
| *Candida albicans* | ATCC 14053 | ATCC, pathogenic | YPD | NA | 30°C |
| *Acinetobacter baumannii* | Ab171 | Lab strain, pathogenic | LB | NA | 37°C |
| *Acinetobacter baumannii* | Ab169 | Lab strain, pathogenic | LB | NA | 37°C |
| *Acinetobacter baumannii* | Ab141 | Lab strain, pathogenic | LB | NA | 37°C |
| *Stenotrophomonas maltophilia* | SM21 | Lab strain, pathogenic | LB | NA | 37°C |
| *Stenotrophomonas maltophilia* | SM3 | Lab strain, pathogenic | LB | NA | 37°C |
| *Stenotrophomonas maltophilia* | D53 | Lab strain, pathogenic | LB | NA | 37°C |
| *Escherichia coli* | BL21 (DE3) | Novagen | LB | NA | 37°C |
